# The University Regents

**Published:** 1906-11

**Authors:** 


The University Regents.—There will be a vacancy on the Board of Regents of The University of Texas, occasioned by the resignation now soon of Hon. J. N. Browning, and Governor-elect Campbell will have to appoint a successor. It is eminently right, proper and just that this successor shall be a physician. It is a shame that there is not a representative of the great medical profession on the Board of Regents. Physicians, generally, are high-class citizens; leaders in every community, as men of learning and high character, exemplars of morals and ethics; and the profession in Texas stands deservedly high. There are in our State Medical Association scores of members who would grace the position and shed honor on it and the State. With a Medical Department of the State University, a school that ranks high and whose graduates are rarely, if ever, turned down by any examining board, but score every time, it is something of a slight and an injustice that there is not a medical man on the Regents' board. Our county medical societies should take the matter up at their November meetings and call upon the Governor by memorial, petition or resolution, to right the wrong, and to extend recognition of the worth, merits and claims of the medical profession by the selection and appointment of one of our many eminent and able members.

